# Atherogenic index of plasma identifies subjects with severe liver steatosis

**DOI:** 10.1038/s41598-025-93141-y

**Published:** 2025-03-17

**Authors:** Carlo De Matteis, Fabio Novielli, Ersilia Di Buduo, Maria Arconzo, Raffaella Maria Gadaleta, Marica Cariello, Antonio Moschetta, Lucilla Crudele

**Affiliations:** 1 Department of Interdisciplinary Medicine, University of Bari “Aldo Moro”, Piazza Giulio Cesare N. 11, 70124 Bari, Italy; 2 INBB National Institute for Biostructure and Biosystems, Via Dei Carpegna, 19 - 00165 Roma, Italia

**Keywords:** Metabolic syndrome, MASLD, Atherosclerosis, Obesity, Diabetes, Mediterranean diet, Hepatology, Endocrine system and metabolic diseases, Metabolic syndrome, Obesity

## Abstract

The Atherogenic Index of Plasma (AIP), calculated by log (Triglycerides/HDL-C), has been proposed as a marker of atherogenic and cardiovascular risk. Atherosclerosis and Metabolic Dysfunction—Associated Steatotic Liver Disease (MASLD) share some pathogenic features and may be considered clinical manifestations of Metabolic Syndrome. In this study, we aimed to investigate the role of increased AIP as a putative metabolic biomarker for MASLD. 1,496 individuals (49% males and 51% females) underwent clinical examination for Metabolic Syndrome at Internal Medicine Division “C. Frugoni” of University Hospital of Bari, Italy in the period between January 2016 and April 2024. Clinical history was recorded, and physical examination, anthropometric measures, biochemical assessment, and abdomen ultrasound were performed. In the overall population, AIP significantly correlated with fasting glycemia (FPG, r = 0.26, p < 0.0001), HbA1c (r = 0.20, p < 0.0001), LDL (r = 0.11, p < 0.0001) and total cholesterol (r = 0.09; p < 0.0001), and anthropometric measures of obesity BMI (r = 0.37, p < 0.0001) and Waist Circumference (r = 0.44; p < 0.001). We then investigated AIP values in patients with and without dysmetabolic conditions, finding that AIP significantly increased as steatosis worsened (p < 0.001). ROC curves identified an optimal cut-off of 0.31 for accurately diagnosing severe steatosis and AIP values above this cut-off discriminated patients with significantly increased (p < 0.0001) fasting glycemia, LDL, and waist circumference, and were strongly associated (p < 0.0001) with MASLD (LLR 85.3), type 2 diabetes (LLR 85.5), abdominal obesity (LLR 72.9), overweight (LLR 151.8), and systemic obesity (LLR 178.4). The risk for being diagnosed with such conditions was found to be even higher in the subpopulation of patients with severe liver steatosis. To validate our findings, we considered another cohort of patients with and without biopsy-proven liver steatosis (public dataset GSE89632), confirming that a significant increase (p < 0.001) in AIP values could be found in patients with liver steatosis compared to healthy controls. AIP can be considered a specific biomarker of fatty liver disease with high sensitivity for the diagnosis of the severe form of liver steatosis. Considering AIP in the evaluation of patients with liver steatosis may augment the accuracy for diagnosing metabolic impairment and MASLD.

## Introduction

The Atherogenic Index of Plasma (AIP), calculated by log (triglycerides/HDL-C), has been validated as a marker of plasma atherogenicity based on its positive association with the lipoprotein particle size, cholesterol esterification rates, and remnant lipoproteinemia^1^. Although it has been studied as a marker of atherogenic risk, increased cardiovascular diseases onset, and Major Cardiovascular Events^2–5^, AIP has also been found inversely correlated with indices of insulin sensitivity^6,7^.

Impairment in glucose metabolism (ranging from glucose intolerance to diabetes), dyslipidemia (i.e. elevated triglycerides and/or reduced HDL-cholesterol serum levels, hypertension and abdominal obesity are recognized as cardiometabolic risk factors. The presence of at least three of these conditions leads to diagnosis of Metabolic Syndrome (MetS) while the coexistence of one or more cardiometabolic risk factors with ultrasound-detected liver steatosis defines Metabolic Dysfunction-Associated Steatotic Liver Disease (MASLD). Although specific genetic predispositions should be considered in MASLD pathogenesis and progression as well as in response to therapeutical interventions^8,9^, MetS and MASLD share common risk factors such as lifestyle choices, dietary habits, excess adiposity and insulin resistance emphasizing the complex interplay of multiple contributors to these conditions.

Early identification of MetS and MASLD in the general population is crucial given their often-asymptomatic nature in the reversible stages and since these conditions are major silent contributors to the development of systemic inflammation, diabetes, cardiovascular diseases, and even cancer^10^. Thus, timely intervention can not only prevent disease progression, but also mitigate the burden of associated life-threatening complications.

In this study, we aimed to investigate the relationship between AIP, MetS, and cardiometabolic risk factors, to explore the putative role of AIP as a potential biomarker for liver steatosis and MASLD.

## Materials and methods

### Setting of the study

We involved 1792 subjects undergoing their first clinical examination for suspected acquired metabolic diseases at Internal Medicine Division “C. Frugoni” of University Hospital of Bari, Italy in the period between January 2016 and April 2024. Each patient received a unique ID and was enlisted in the electronic health register of Metabolic Diseases of the Department of Interdisciplinary Medicine at “Aldo Moro” University of Bari. Patients with a history of alcohol abuse, viral hepatitis, acute heart diseases (cardiac failure, coronary arterial disease, acute arrhythmias), renal and hepatic failure, active infections, and neoplastic diseases with recent onset (less than 10 years) and/or under chemotherapy were excluded. Thus, the final study was conducted on a population 1,496 adults (49% males and 51% females).

### Clinical and biochemical assessment

Physical examination, anthropometric measures, biochemical assessment, and abdomen ultrasound were performed. Average systolic and diastolic blood pressure (BP) were recorded for each patient in three different measurements using a manual sphygmomanometer. Hypertension was diagnosed for systolic BP ≥ 130 mmHg, diastolic BP ≥ 85 mmHg and/or treatment with antihypertensive agents. Anthropometric assessment was performed using standardized procedures. Briefly, waist circumference (WC) was measured at the midpoint between the inferior part of the 12th costa and the anterior–superior iliac crest. Body Mass Index (BMI) was computed as weight (Kg) divided by the height squared (sqm) and subjects were defined overweight for BMI values between 25 kg/sqm and 29.9 kg/sqm and obese for values above 30 kg/sqm. Morning blood samples were obtained after 12 h of fasting from the antecubital veins. After blood clotting and centrifugation, serum was processed for analysis of biochemical markers of lipid and glucose metabolism. Renal, liver, inflammation thyroid function markers were as well studied following standardized biochemical procedures. All biochemical measurements were centralized and performed in the ISO 9001 certified laboratories of the University Hospital of Bari. Specifically, a complete blood count with determination of leukocyte’s subpopulation was performed. Measurements of total and HDL cholesterol, FPG, triglycerides were obtained through enzymatic colorimetric assay (Siemens, Erlangen, Germany). 25-OH vitamin D was determined by CLIA on the LIAISON analyzer (DiaSorin, Inc., Stillwater, MN, USA). HbA1c was assessed in human whole blood using ion-exchange high-performance liquid chromatography (HPLC) on the Bio-Rad Variant II Hemoglobin A1c Program (BIO-RAD Laboratories Srl, Milan, Italy).

AIP was calculated as the logarithmically transformed ratio of molar concentrations of Triglycerides (TG) to High Density Lipoprotein (HDL)-cholesterol^2^.

Patients were also asked to answer specific questions related to the Chrono Med Diet Score (CMDS), a questionnaire we previously validated to assess adherence to Mediterranean Diet (MedDiet) and lifestyle, with CMDS score below 15 indicating poor adherence^11^. All questionnaires were administered in a row with standard operating procedures by trained personnel.

MetS was diagnosed according to the International Diabetes Federation (IDF) definition^12^ and abdominal obesity was defined for WC values equal or above 80 cm in women and 94 cm in men. Type 2 Diabetes (T2D) was diagnosed according to international criteria^13^: HbA1c (percentage of glycosylated haemoglobin) ≥ 6.5% and/or fasting plasma glucose (FPG) ≥ 126 mg/dl and/or ongoing treatment for diabetes. Unfortunately, 2-h Plasma Glucose (PG) during 75-g Oral Glucose Tolerance Test was not performed.

After an overnight fasting, patients underwent an abdominal ultrasound scanning performed by two expert physicians with more than 10 years of experience in ultrasonography with a 3.5–5 MHz convex probe (Esaote My Lab 70 Gold ultrasound system). B-mode ultrasound was used for assessment of fatty liver. Mild steatosis was defined when a mild diffuse increase in fine echoes in the hepatic parenchyma was found but normal visualisation of the diaphragm and intrahepatic vessel borders was still possible. Moderate steatosis was represented by a moderate diffuse increase in fine echoes with slightly impaired visualisation of the intrahepatic vessels and diaphragm. Severe steatosis was diagnosed when a marked increase in fine echoes with poor or no visualisation of the intrahepatic vessel borders, diaphragm and posterior portion of the right lobe of the liver were present^14^. MASLD diagnosis was based on the presence of liver steatosis identified by ultrasound and at least one out of five cardiometabolic risk factors^15^.

### Data analysis

Shapiro–Wilk Test was used to assess normal distribution. Descriptive statistical analyses of the study sample were performed, and results were expressed as mean ± standard deviation (SD) for numerical data, in counts and percentages for categorical data. Student T-test was performed for comparisons between two groups, while one-way ANOVA was performed for comparisons among more than two groups. Correlations between AIP and main metabolic biomarkers were analysed and estimated using Pearsons’s correlation coefficient (r). The receiver–operating characteristic (ROC) curves were used to determine the optimum cut-off values of AIP in discriminating hepatic steatosis and MetS. Empirical ROC curves were plotted along with a calculation of the area under the curve (AUC) with 95% confidence intervals (C.I.) and one-sided upper p-values (p) for the null hypothesis AUC = 0.5. Youden’s Index, or equivalently, the highest Sensitivity + Specificity, was used to determine optimal cut-offs. Contingency tables, Chi-squared test, and Fisher’s exact test if indicated, were used to study the association between pathologic values of AIP and dysmetabolic conditions. The results were expressed as Odds Ratios with their relative 95% confidence interval (95% CI) and were graphically plotted in a forest plot; logarithmic-likelihood ratios (LLR) were also reported. p < 0.05 were considered statistically significant. All analyses were performed using the NCSS 12 Statistical Software, version 12.0.2018 (NCSS, LLC Company, Kaysville, UT, USA) and GraphPad Prism, version 10 (GraphPad Software; San Diego, CA, USA).

## Results

### Baseline characteristics of study population

The study population consisted of 735 males and 761 females, with a mean age of 57.2 ± 14.6 years. According to BMI, 36% of subjects were normal weight, 36% were overweight and 28% obese. A high prevalence of abdominal obesity (78%) was observed based on WC, with a mean WC of 97.8 ± 14.8 cm, exceeding the threshold for MetS and MASLD diagnoses. Overall, 770 patients (51%) had MetS and 567 patients (38%) were affected by T2D. Mean CMDS value (10.9 ± 4.7) was indicative of low adherence to MedDiet, and both Framingham Score (18.2 ± 16.9%) and Score 2/OP (10.9 ± 4.7%) depicted a condition of intermediate-to-high cardiovascular risk. Liver steatosis was diagnosed via ultrasound in 900 patients (60%) and specifically, 508 (34%) had mild steatosis, 271 (18%) had moderate steatosis and 121 (8%) had severe steatosis. MASLD was diagnosed in 881 patients (59%). Table 1 summarises all baseline characteristics of the population.

**Table 1:** Study population characterization (N = 1,496).

Males (n)	735 (49%)
Age (Years)	57.2 ± 14.6
Waist Circumference (cm)	97.8 ± 14.8
BMI (Kg/Sqm)	27.4 ± 5.6
FPG (mg/dL)	100.4 ± 29.1
HbA1c (mmol/mol)	41.1 ± 11.2
AST (U/L)	23.8 ± 10.4
ALT (U/L)	30.3 ± 18.3
GGT (U/L)	33.9 ± 36.4
Total Cholesterol (mg/dL)	181.4 ± 40.7
HDL Cholesterol (mg/dL)	53.8 ± 14.4
LDL Cholesterol (mg/dL)	105.8 ± 34.7
Triglycerides (mg/dL)	118.1 ± 68.1
25-OH vitamin D (ng/ml)	23.9 ± 13.8
CMDS	10.9 ± 4.7
AIP	0.30 ± 0.28
BMI = 25–29.9 kg/sqm (n)	535 (36%)
BMI ≥ 30 kg/sqm (n)	417 (28%)
Abdominal obesity (n)	1167 (78%)
Metabolic syndrome (n)	770 (51%)
Type 2 diabetes (n)	567 (38%)
Liver steatosis (n)	900 (60%)
Mild (n)	508 (34%)
Moderate (n)	271 (18%)
Severe (n)	121 (8%)
MASLD (n)	881 (59%)

Data are reported as mean ± SD (Standard Deviation) for quantitative variables and in count and percentage for categorical variables. BMI ≥ 25 kg/sqm depicts a condition of overweight, while BMI ≥ 30 kg/sqm a condition of obesity. Abdominal obesity was diagnosed for Waist Circumference values ≥ 80 cm in females and ≥ 94 cm in males. Metabolic Syndrome was diagnosed when subjects had increased waist circumference plus at least two other criteria among hyperglycaemia, low HDL, hypertriglyceridemia, and hypertension. Type 2 Diabetes was diagnosed for FPG > 126 mg/dl or HbA1c > 6.4% or ongoing anti-diabetic treatment. Liver steatosis was assessed through Abdomen Ultrasound. Mild steatosis is represented by a mild diffuse increase in fine echoes in the hepatic parenchyma with normal visualisation of the diaphragm and intrahepatic vessel borders. Moderate steatosis is represented by a moderate diffuse increase in fine echoes with slightly impaired visualisation of the intrahepatic vessels and diaphragm. Severe steatosis is represented by a marked increase in fine echoes with poor or no visualisation of the intrahepatic vessel borders, diaphragm, and posterior portion of the right lobe of the liver. MASLD diagnosis was based on the presence of liver steatosis and at least one out of five cardiometabolic risk factors.

*BMI* body mass index, *FPG* fasting plasma glucose, *HbA1c* glycosylated haemoglobin, *AST* aspartate transaminase, *ALT* alanine transaminase, *GGT* gamma-glutamyl transferase, *CMDS* chrono med diet score, *AIP* atherogenic index of plasma, *MASLD* metabolic dysfunction-associated steatotic liver disease.

### Correlations between AIP and features of MetS and MASLD

**Fig. 1:**
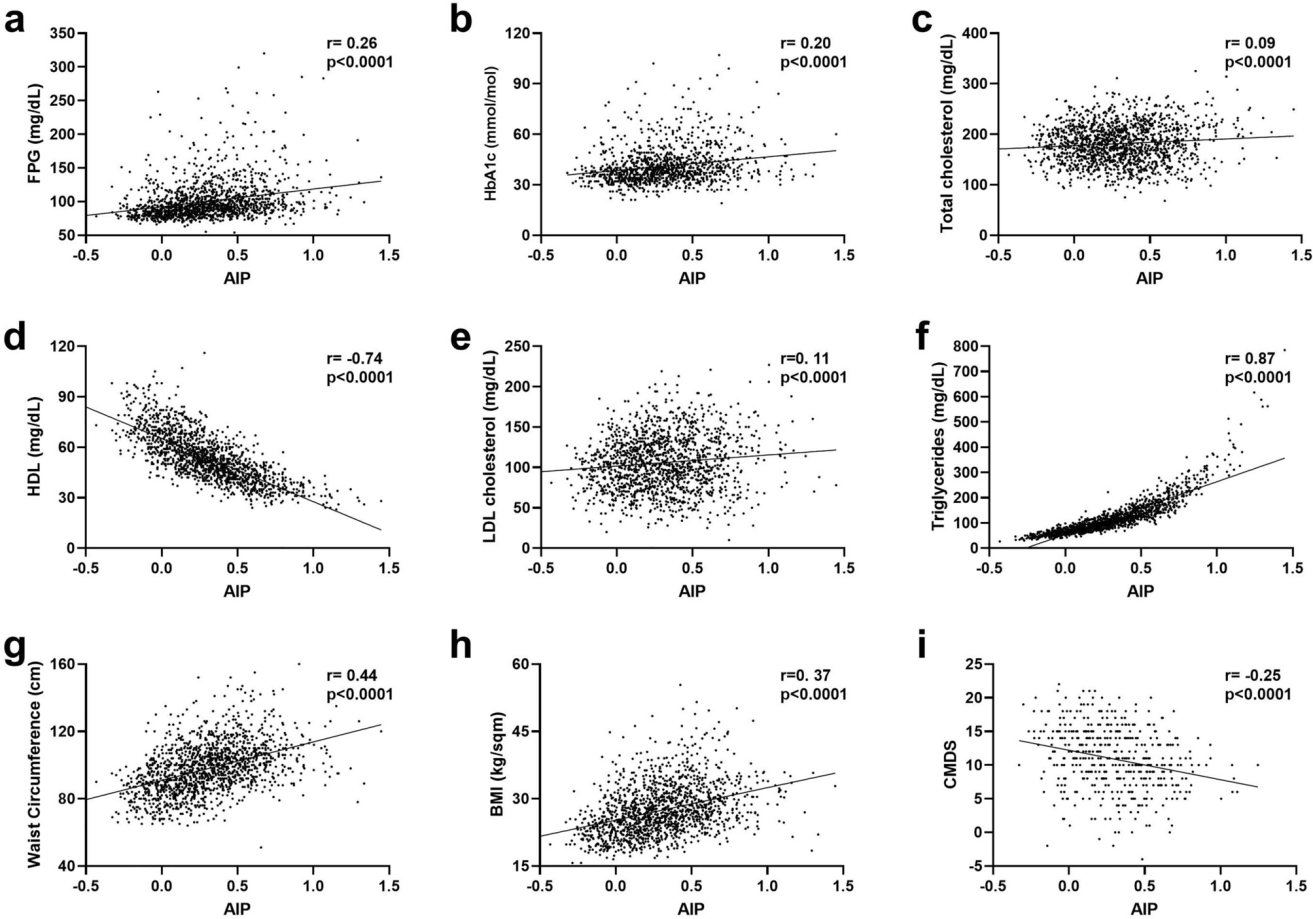
Correlations between AIP and main metabolic biomarkers. AIP Pearson’s correlations (r) and p-values (p) with FPG (**a**), HbA1c (**b**), Total Cholesterol (**c**), HDL Cholesterol (**d**), LDL Cholesterol (**e**), Triglycerides (**f**), Waist circumference (**g**), BMI (**h**), CMDS (**i**) are reported. *AIP* atherogenic index of plasma, *FPG* fasting plasma glucose, *HbA1c* glycosylated haemoglobin, *BMI* body mass index, *CMDS* chrono med diet score.

To assess whether AIP could represent a reliable index of the overall metabolic status, we performed a study correlation between AIP and main metabolic biomarkers (Fig. 1).

Considering the glycaemic profile, AIP showed a significant direct correlation with both FPG (Fig. 1a; p < 0.0001) and HbA1c (Fig. 1b; p < 0.0001), with coefficients of 0.26 and 0.20, respectively.

In view of these relationships with bio-humoral metabolic biomarkers, we then studied if also anthropometric measures of adiposity significantly correlated with AIP values, finding that AIP positively correlated with both WC (Fig. 1g; p < 0.0001) and BMI (Fig. 1h; p < 0.0001); in line with this, an inverse association between AIP and adherence to MedDiet assessed through CMDS (Fig. 1i; p < 0.0001) was observed.

### Comparisons of AIP values in patients with and without dysmetabolic conditions

**Fig. 2:**
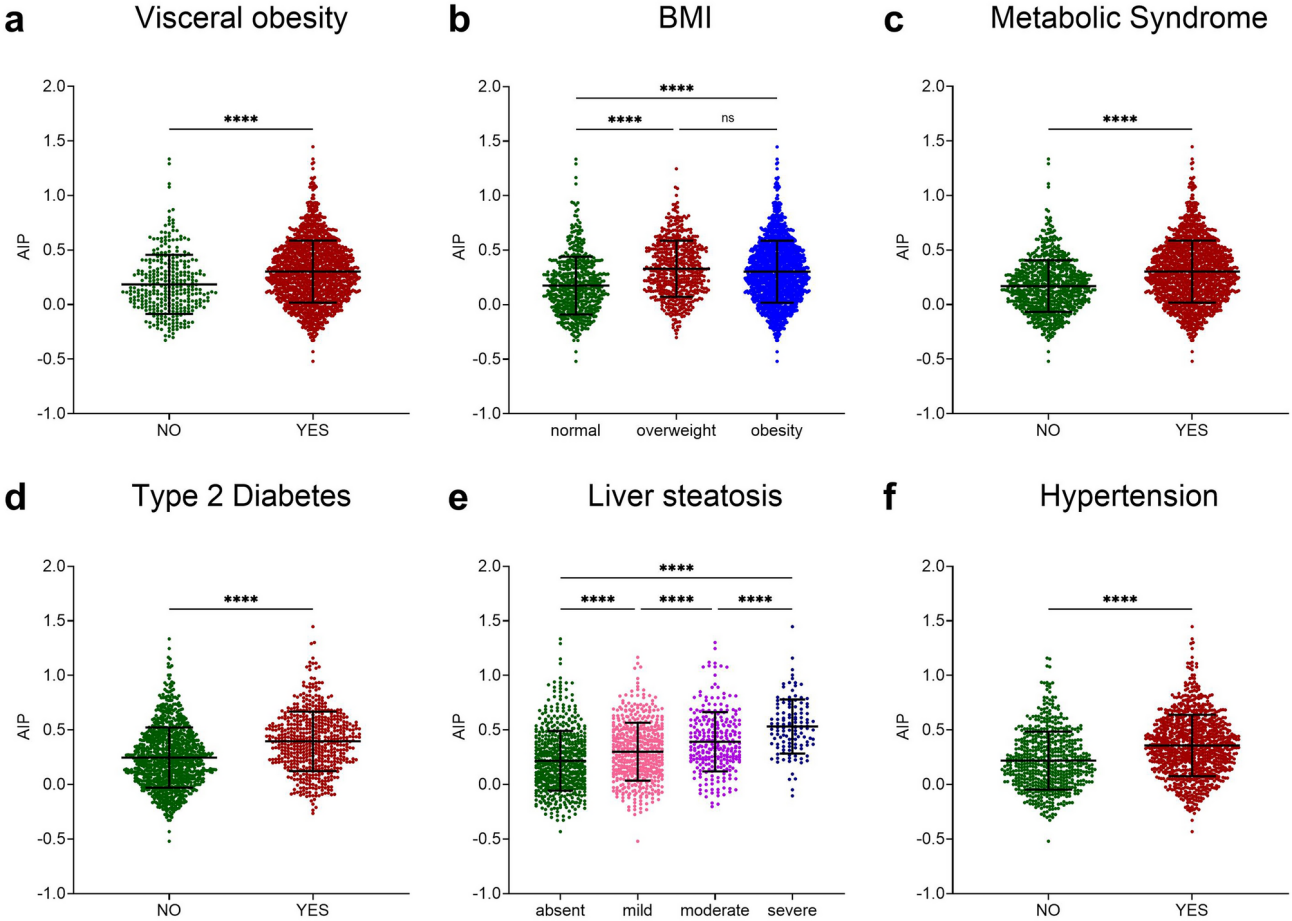
Comparisons of AIP values in patients with and without dysmetabolic conditions. The scatter plots show the mean and SD with each point representing one observation. Student T-test was performed for comparisons between two groups, while One-way ANOVA was used for comparisons among more than two groups. Statistical significance was assessed for p-values (p) < 0.05; ****p < 0.0001. (**a**) Abdominal obesity was diagnosed for Waist Circumference values ≥ 80 cm in females and ≥ 94 cm in males. (**b**) BMI ≥ 25 kg/sqm depicts a condition of overweight, while BMI ≥ 30 kg/sqm a condition of obesity. (**c**) Metabolic Syndrome was diagnosed when subjects had increased waist circumference plus at least two other criteria among hyperglycaemia, low HDL, hypertriglyceridemia, and hypertension. (**d**) Type 2 Diabetes was diagnosed for FPG > 126 mg/dl or HbA1c > 6.4% or ongoing anti-diabetic treatment. (**e**) Liver steatosis was assessed through Abdomen Ultrasound. Mild steatosis is represented by a mild diffuse increase in fine echoes in the hepatic parenchyma with normal visualisation of the diaphragm and intrahepatic vessel borders. Moderate steatosis is represented by a moderate diffuse increase in fine echoes with slightly impaired visualisation of the intrahepatic vessels and diaphragm. Severe steatosis is represented by a marked increase in fine echoes with poor or no visualisation of the intrahepatic vessel borders, diaphragm, and posterior portion of the right lobe of the liver. (**f**) Hypertension was assessed for systolic arterial blood pressure (SAP) ≥ 130mmHg and/or diastolic arterial blood pressure (DAP) ≥ 85mmHg and/or treatment with antihypertensive agents. *BMI* Body Mass Index; *ns* not-significant.

In view of above-reported AIP correlations, we then compared AIP values in patients with and without dysmetabolic conditions with the aim of analysing the presence of possible variations according to the occurrence of certain metabolic alterations (Supplementary Table 1; Fig. 2). Patients with abdominal obesity (Fig. 2a) exhibited significantly higher AIP values compared to those with normal WC (p < 0.0001). Similarly, individuals with normal weight demonstrated lower mean API values than both overweight (p < 0.0001) and obese (p < 0.0001) patients when stratified for BMI. However, no significant AIP difference was found between overweight and obese groups (Fig. 2b). Furthermore, patients with MetS (Fig. 2c; p < 0.0001), T2D (Fig. 2d; p < 0.0001), and Hypertension (Fig. 2e; p < 0.0001) displayed significantly elevated AIP levels compared to their counterparts not affected by these conditions.

Regarding liver steatosis, not only mean AIP values were significantly higher (p < 0.0001) in patients with liver steatosis (0.36 ± 0.28) compared to those without (0.22 ± 0.27), but AIP values also increased progressively with the severity of steatosis (Fig. 2f). These findings suggest that increased AIP may serve as a potential discriminator for patients with dysmetabolic conditions.

To validate our findings, we calculated AIP in another cohort of patients with and without biopsy-proven liver steatosis, using the public-available data derived from the gene expression omnibus (GEO) database. Analysing the public dataset GSE89632^16,17^, we detected a significant increase (p < 0.001) in AIP values in patients with liver steatosis compared to healthy controls (Supplementary Fig. 1).

### AIP optimal cut-offs for diagnosis of metabolic syndrome and MASLD

**Fig. 3:**
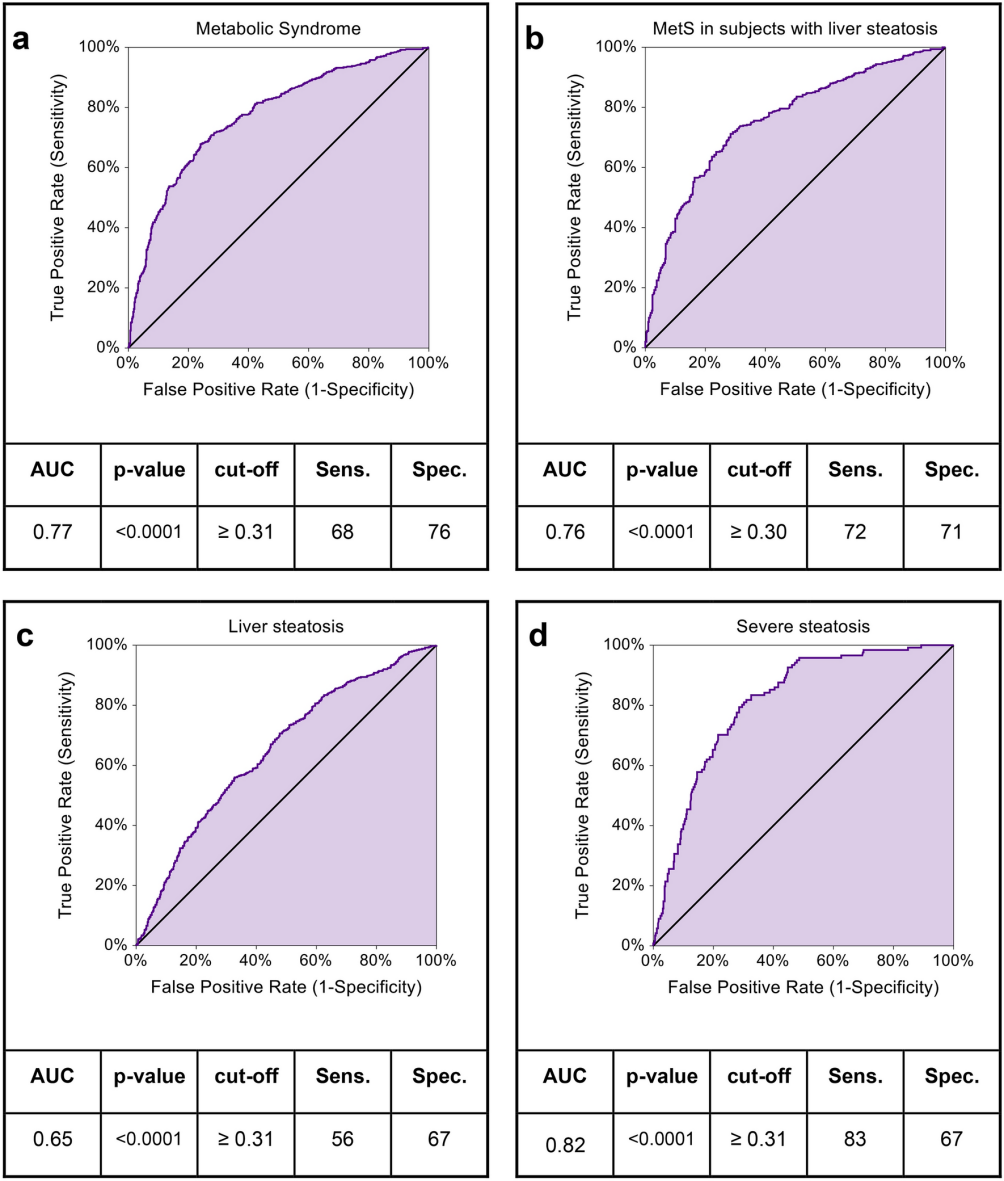
ROC curves of AIP in prediction of liver steatosis and Metabolic Syndrome. Empirical ROC curves of AIP for prediction of Metabolic Syndrome in the overall population (**a**) and in subjects with liver steatosis (**b**), of liver steatosis (**c**), and severe liver steatosis (d). Area under curve (AUC) and p-value together with calculated cut-off and respective Sensitivity and Specificity are reported for each condition. Statistical significance was assessed for p-values (p) < 0.05.

To determine optimal AIP cut-off values for diagnosing MetS and MASLD, ROC curve analyses were conducted (Fig. 3).

AIP ROC curve yielded a cut-off value of 0.31 in discriminating patients with MetS, proving a good performance (AUC = 0.77, p < 0.0001, sensitivity = 68% and specificity = 76%) (Fig. 3a). Among patients with MetS and liver steatosis, this cut-off slightly decreased (0.30) with an improved Youden’s index (sensitivity = 72%; specificity = 71%), and a similar AUC (0.76, p < 0.0001) (Fig. 3b). When considering liver steatosis alone, the ROC curve showed an AUC of 0.65 (p < 0.0001) and optimal cut-off of 0.31 (sensitivity = 56%, specificity = 67%) (Fig. 3c). Notably, this cut-off of AIP also discriminated severe steatosis efficiently (AUC = 0.82, p < 0.0001, Youden’s Index sensitivity = 83%, specificity = 67%) (Fig. 3d), These findings underscore AIP’s ability to accurately distinguish dysmetabolic conditions, with a constantly improving performance as steatosis severity increases.

### Risk of dysmetabolic conditions according to AIP values

**Fig. 4:**
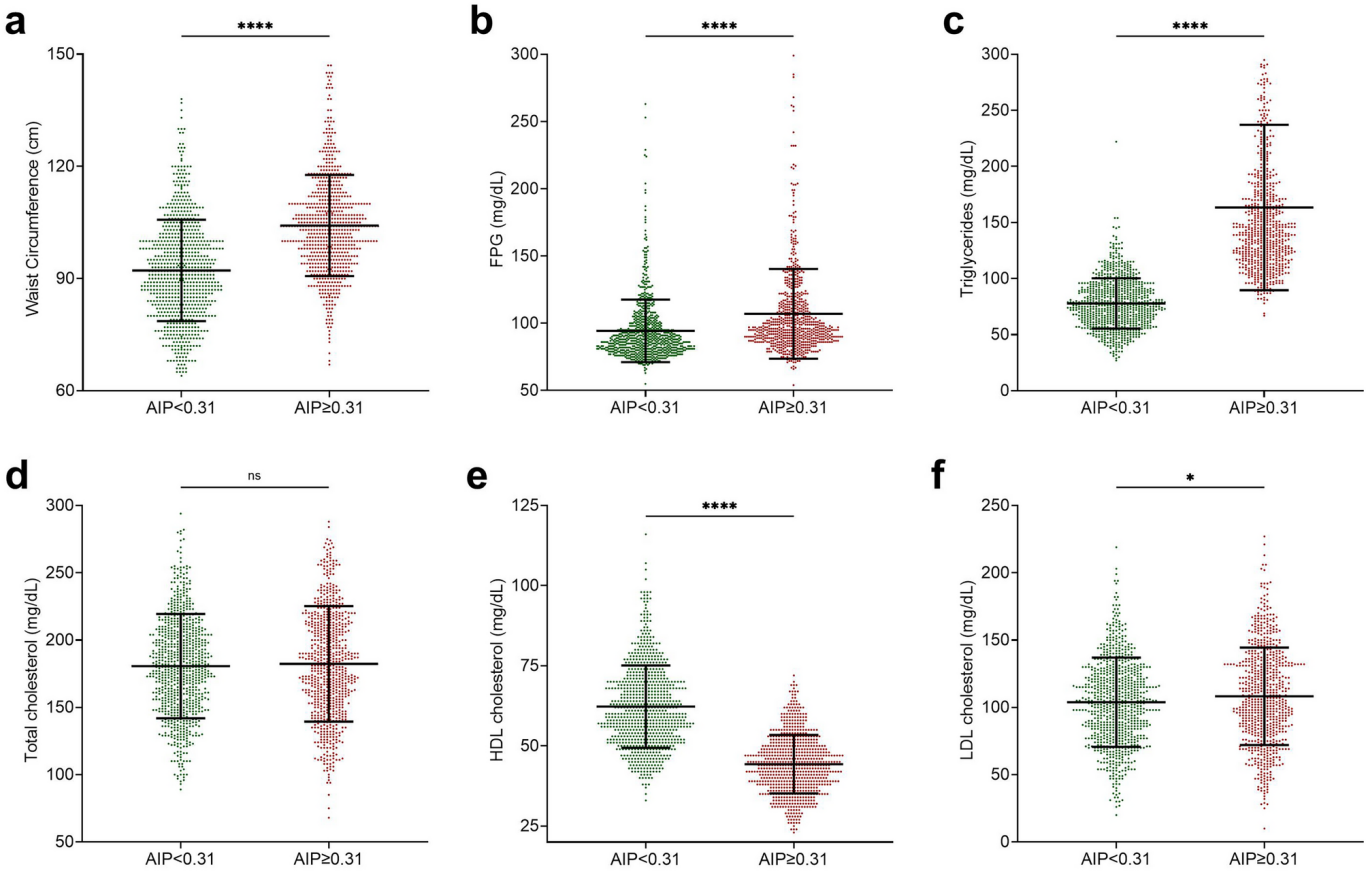
Comparisons of metabolic biomarkers in patients with AIP values over and below the cut-off value of 0.31. The scatter plots show the mean and SD with each point representing one observation The scatter plots show the mean and SD with each point representing one observation. Student T-test was performed for comparisons between two groups. Statistical significance was assessed for p-values (p) < 0.05; *p < 0.05; ****p < 0.0001. *AIP* atherogenic index of plasma, *FPG* fasting plasma glucose, *ns* not significant.

Furthermore, to determine if AIP values exceeding the previously established cut-off of 0.31 were associated with specific metabolic diseases, we compared main metabolic biomarkers in subgroups of patients stratified for AIP value (Fig. 4). We found that patients with AIP ≥ 0.31 showed significantly higher WC (92.2 vs. 104.2; p < 0.0001), FPG (94.4 vs 107; p < 0.0001), triglycerides (77.8 vs. 163.4, p < 0.0001), and LDL cholesterol (103.8 vs. 108.2; p < 0.05), as well as reduced HDL cholesterol (62.3 vs. 44.3; p < 0.0001). No significant difference was found in total cholesterol value (180.6 vs 182.3, not significant).

**Fig. 5:**
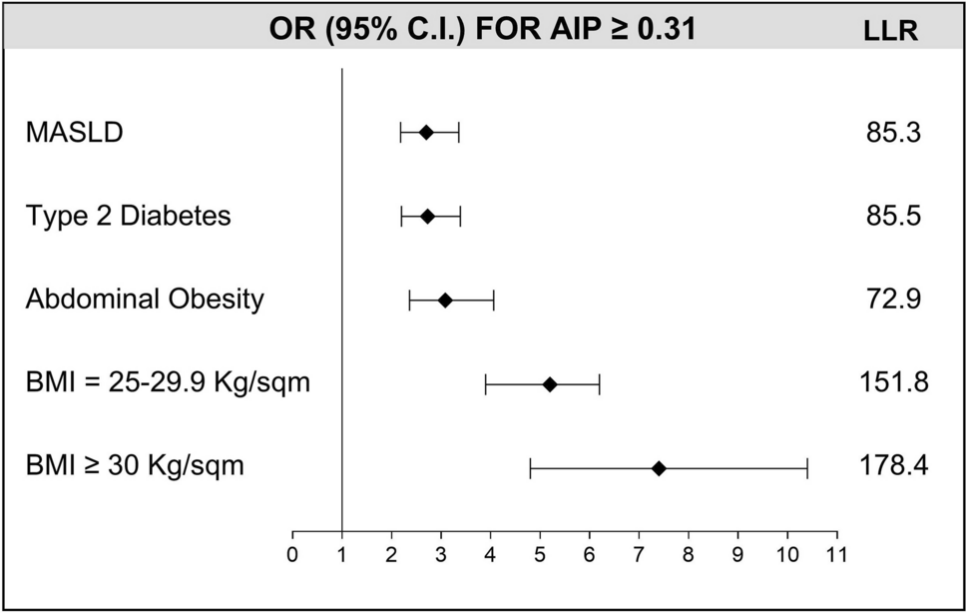
ORs of being diagnosed with dysmetabolic conditions when AIP ≥ 0.31. ORs with their 95% Confidence Interval are represented. All the conditions were significantly associated (p < 0.0001) to increased AIP. Also log-likelihood ratios (G-squared) are reported. *OR* odd ratio, *AIP* atherogenic index of plasma, *MASLD* metabolic-dysfunction associated steatotic liver diseases, *BMI* body mass index.

A chi-square analysis revealed a strong association (p < 0.0001) between elevated AIP and all studied metabolic conditions. OR for MASLD was 2.7 (95% C.I. 2.2–3.4) with LLR of 85.3. Similarly, T2D showed an OR of 2.7 (95% C.I. 2.2–3.4) and LLR = 85.5. OR values increased for abdominal adiposity assessed by WC (OR 3.1; 95% C.I. 2.4–4.1) with LLR of 72.9, but was even higher (OR 5.2, 95% C.I. 3.9–6.2) for BMI ≥ 25 kg/sqm with LLR = 151.8 and BMI ≥ 30 kg/sqm (OR 7.4, 95% C.I. 4.8–10.4, LLR 178.4) (Fig. 5).

Since data from our study show that AIP is strictly associated with severe steatosis, we performed the same chi-square analysis with odds ratios only in patients with severe steatosis (Table 2). Results confirmed that these subjects are at risk of being diagnosed with these cardiometabolic conditions. Indeed, OR for MASLD increased to 4.3 (95% C.I. 3.0–5.2) with LLR of 151.1; OR for T2D was 3.9 (95% C.I. 2.8–4.9) with LLR = 129.4. OR value for abdominal adiposity assessed by WC (OR 5.5; 95% C.I. 4.1–9.4) also increased with LLR of 134.8 and was even higher for overweight (OR 7.1, 95% C.I. 5.0–10.4, LLR = 173.2) and systemic obesity (OR 9.5, 95% C.I. 7.5–13.2, LLR = 193.2). These results underscore the utility of AIP specifically in severe steatosis.

**Table 2:** ORs of being diagnosed with MASLD and cardiometabolic conditions when AIP ≥ 0.31 in individuals with severe steatosis.

Parameter	OR	95% C.I	LLR	p-value
MASLD	4.3	3.0–5.2	151.1	< 0.0001
TYPE 2 DIABETES	3.9	2.8–4.9	129.4	< 0.0001
ABDOMINAL OBESITY	5.5	4.1–9.4	134.8	< 0.00001
BMI ≥ 25 kg/sqm	7.1	5.0–10.4	173.2	< 0.00001
BMI ≥ 30 kg/sqm	9.5	7.5–13.2	193.2	< 0.00001

*OR* odd ratio, *C.I.* confidence interval, *LLR* log-likelihood ratio, *p* p-value, *AIP* atherogenic index of plasma, *MASLD* metabolic-dysfunction associated steatotic liver diseases, *BMI* body mass index.

## Discussion

In this study we investigated the role of AIP, an index of atherogenic and cardiovascular risk, in the context of liver steatosis, finding that it could be considered a robust biomarker for identifying and stratifying patients at risk of MASLD and its associated metabolic complications, particularly when severe liver steatosis is present. Previous studies have evaluated AIP as marker of undiagnosed diabetes^18^ and risk of prediabetes progression^19^, while many others have studied the role of non-invasive scores in the diagnosis of liver steatosis and fibrosis^20,21^. Furthermore, AIP had been proposed as a good and independent predictor for Metabolic dysfunction-Associated Fatty Liver Disease (MAFLD) in patients with T2D^22^ and as a reliable biomarker for the diagnosis of Non-alcoholic fatty liver disease (NAFLD)^23^. Nonetheless, to the best of our knowledge, this is the first study to evaluate AIP within the framework of the updated MASLD definition^15^. We observed significant AIP correlations not only with biomarkers of lipid metabolism, but also with fasting glycemia, HbA1c, WC and BMI, reflecting its link with both dyslipidaemia and obesity. These results emphasize the intricate relationship between dyslipidaemia and adiposopathy, the wide spectrum of conditions associated to adipocytes enlargement due to excessive energy storage that leads to dysfunctional adipose tissue, the source of inflammatory cytokines and hormonal signalling boosting low-grade systemic inflammation and insulin resistance, the hallmarks of obesity and diabetes^24,25^. Coherently, in our cohort the role of energy excess due to unhealthy dieting is demonstrated by the significant inverse correlation between adherence to MedDiet and AIP values.

In addition to obesity, MetS, and T2D, in our study population AIP values were found to be significantly increased also in patients with liver steatosis. These results align with previous findings in a large US cohort demonstrating a strong association between AIP and hepatic steatosis, suggesting that AIP may be used as an efficient measure for clinical prediction of fatty liver^26^.

Notably, our study extended these findings by revealing that AIP detects severe steatosis.

While a higher cut-off of 0.62 has been proposed for predicting MAFLD in patients with T2D^27^ and as an independent biomarker associated with plaque instability^28^, our study identified a lower cut-off of 0.30 for detecting liver steatosis. This discrepancy suggests a potential underdiagnosis of cardiometabolic risk and MASLD based on existing thresholds. Furthermore, the association between elevated AIP and both abdominal and general obesity, as well as T2D, highlights its potential role in monitoring disease progression and preventing metabolic complications.

From a biological standpoint, the strength of AIP in capturing not only atherosclerosis conditions but also MASLD may lie in its formula that considers both TG and HDL. HDL particles protect from atherosclerosis since they are involved in the Reverse Cholesterol Transport (RCT) pathway, through which dietary cholesterol is transported from peripheral tissues to the liver where it can be secreted in bile feces directly or after its conversion into bile salts. Thus, HDL cholesterol is thus considered ‘good’ cholesterol because it removes cholesterol from the periphery^29^. On a gene transcriptional level, the scenario is orchestrated by the cholesterol-oxysterol sensor, nuclear receptor Liver X Receptor (LXR)^30^. Indeed, elevated cholesterol levels activate the LXR transcriptional pathway, leading to decreased cholesterol absorption and cellular uptake. Simultaneously, this pathway stimulates fatty and bile acid biosynthesis, promoting cholesterol excretion, and fecal disposal through HDL-triggered RCT. Our study group also previously demonstrated that in the immediate post-infarction period there is a spontaneous ligand-induced activation of the LXR driven cholesterol efflux capacity of intracoronary monocytes to overcome the reduced serum ability to accept cholesterol and to inhibit the post-infarction proinflammatory local microenvironment^31^. Unfortunately, systemic LXR activation concurrently promotes hepatic de novo lipogenesis, hepatic steatosis, and hypertriglyceridemia via direct activation of the SREBP-1c and fatty acids (FAs) synthesis pathways, resulting in a rise in VLDL TG^29,32,33^. Indeed, TG circulating levels are determined by the balance between their production and clearance. Since d*e novo* synthesis of FAs and TG from dietary carbohydrates mainly occurs in the liver, about 25% of the hepatic FAs that accumulate in steatotic condition are furnished by the hepatic de novo lipogenesis and the FA processing pathways^34^. These pathways are mainly controlled by PPARα^35^ and SREBP-1c^36^, whose expression is induced by insulin, which explains the classic ability of insulin to enhance the conversion of glucose to FAs^37,38^. In subjects with insulin-resistance, pancreatic insulin secretion is increased, both in the fasting and postprandial phases, since peripheral tissues require higher concentrations of insulin for glucose uptake^39,40^. Furthermore, these findings underscore the huge role of dietary habits and lifestyle in determining metabolic impairment and liver steatosis, as also shown by the inverse correlation between adherence to MedDiet and AIP values in our population.

In this view, our study highlights the coherence of AIP in identifying not only the single major dysmetabolic conditions associated with adiposopathy such as obesity and diabetes, but also to assess the severity of hepatic involvement.

Some limitations of the present study need to be highlighted. Firstly, the predominantly European study population necessitates caution when generalizing findings to diverse populations. Secondly, the cross-sectional study design precludes evaluation of AIP changes over time, necessitating longitudinal studies to assess its utility for patient monitoring. Thirdly, the potential impact of lipid-lowering medications on AIP levels should be investigated further, as these treatments could influence AIP values and potentially affect its diagnostic accuracy. Lastly, 2-h PG during 75-g OGTT test was not performed thus diabetes and MASLD could have been underdiagnosed.

## Conclusions

In conclusion, AIP can be considered a specific biomarker of fatty liver disease with high sensitivity for the diagnosis of severe liver steatosis. Considering AIP in the evaluation of patients with liver steatosis may augment the accuracy for diagnosing metabolic impairment and MASLD. Thus, since AIP should be elected as a *bona fide* biomarker of risk not only for atherosclerosis but also for severe liver steatosis, AIP evaluation may facilitate timely interventions to prevent or mitigate the progression of MASLD and related comorbidities.

## Supplementary Information

Supplementary Figure 1.

Supplementary Table 1.

## Data Availability

Data presented in this study are available on request from the corresponding author.

## Abbreviations

AIP: Atherogenic index of plasma
MetS: Metabolic syndrome
MASLD: Metabolic dysfunction-associated steatotic liver disease
BP: Blood pressure
WC: Waist circumference
BMI: Body mass index
TG: Triglycerides
CMDS: Chrono med diet score
MedDiet: Mediterranean diet
IDF: International Diabetes Federation
T2D: Type 2 diabetes
HbA1c: Percentage of glycosylated haemoglobin
FPG: Fasting plasma glucose
CVD: Cardiovascular disease
SD: Standard deviation
ROC: Receiver–operating characteristic
AUC: Area under the curve
C.I.: confidence intervals
p: P-values
LLR: Logarithmic-likelihood ratio
GEO: Gene expression omnibus
MAFLD: Metabolic dysfunction-associated fatty liver disease
NAFLD: Non-alcoholic fatty liver disease
RCT: Reverse cholesterol transport
LXR: Liver X receptor
FAs: Fatty acids
