# Supplementary material for: Atherogenic index of plasma identifies subjects with severe liver steatosis
Source: Sci Rep. 2025 Mar 17;15:9136. doi: 10.1038/s41598-025-93141-y (PMC11914574; doi:10.1038/s41598-025-93141-y)
Supplement: Supplementary file 1 — Supplementary Figure 1. [file 41598_2025_93141_MOESM1_ESM.docx]

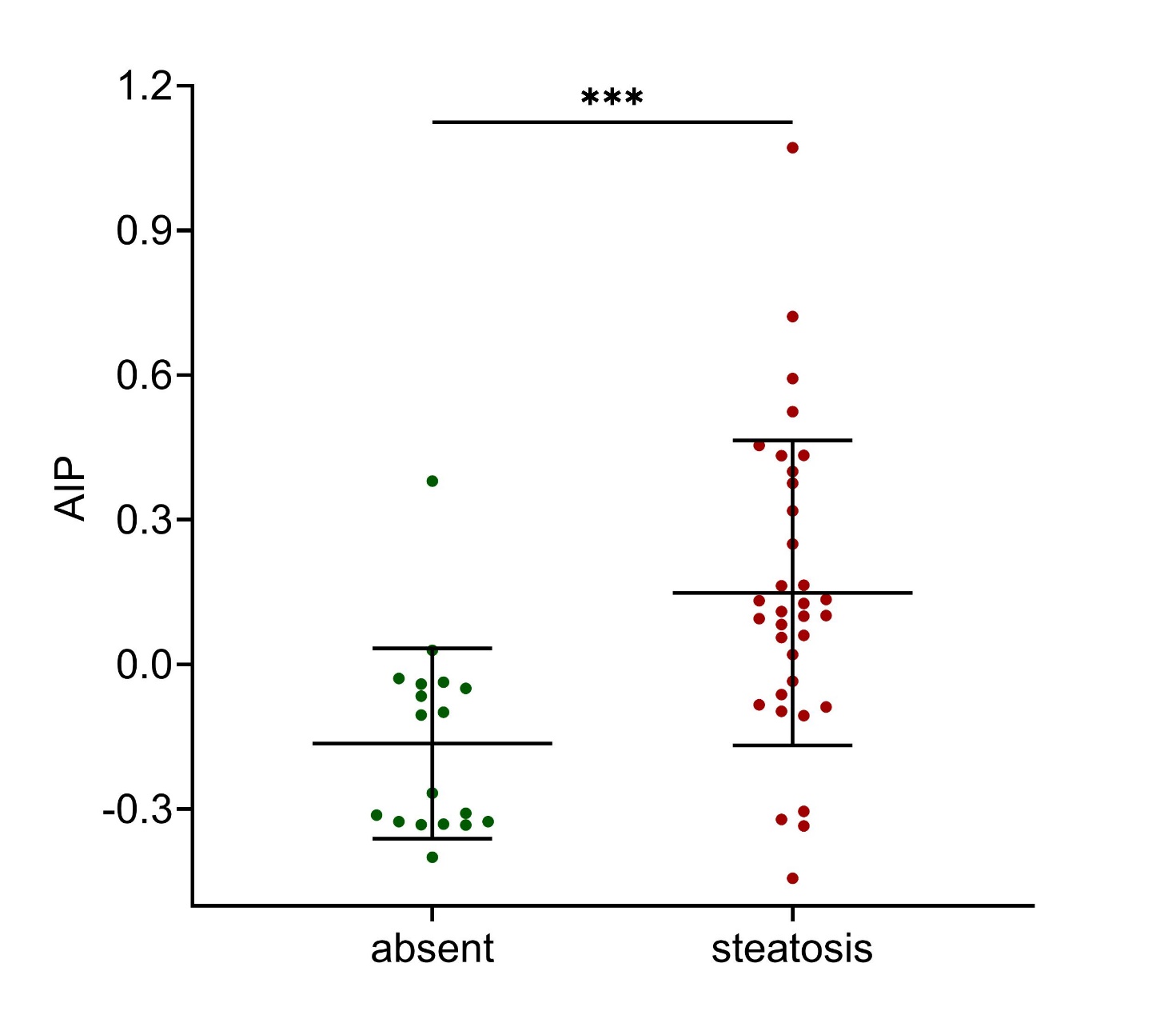


**Supplementary figure 1.** **Comparison of AIP values in a validation cohort.**

Comparison of AIP values in patients with (n=34) and without (n=18) liver steatosis from public dataset GSE89632. The dataset used in this study was identified through a targeted search in the GEO database using the keywords: 'NAFLD' AND 'healthy controls' AND 'HDL'. The search returned two results: one related to mouse data and one related to human data, then the human dataset, corresponding to GEO accession number GSE89632, was selected. This dataset includes triglyceride and HDL cholesterol levels for subjects with steatosis and healthy controls. These parameters were essential for calculating the AIP, which was calculated using the formula TG/HDL-C. Student T-test was performed, statistical significance was assessed for p-values (p)<0.05. ***p<0.001
