# Supplementary material for: Atherogenic index of plasma identifies subjects with severe liver steatosis
Source: Sci Rep. 2025 Mar 17;15:9136. doi: 10.1038/s41598-025-93141-y (PMC11914574; doi:10.1038/s41598-025-93141-y)
Supplement: Supplementary file 2 — Supplementary Table 1. [file 41598_2025_93141_MOESM2_ESM.docx]

**Supplementary table 1.** **Comparisons of AIP values in patients with and without dysmetabolic conditions.**

|  | NO | | | YES | | | | p-value | |
| --- | --- | --- | --- | --- | --- | --- | --- | --- | --- |
| Abdominal Obesity | 0.19±0.27 | | | 0.30±0.28 | | | | <0.0001 | |
| Type 2 Diabetes | 0.25±0.28 | | | 0.40±0.27 | | | | <0.0001 | |
| Metabolic Syndrome | 0.17±0.24 | | | 0.30±0.28 | | | | <0.0001 | |
| Hypertension | 0.22±0.27 | | | 0.36±0.28 | | | | <0.0001 | |
| Liver Steatosis | 0.22±0.27 | | | 0.36±0.28 | | | | <0.0001 | |
|  |  | | |  | | | |  | |
|  | **NO** | **MILD** | | | **MODERATE** | | **SEVERE** | | **p-value** |
| Liver Steatosis grades | 0.22±0.27 | 0.30±0.27 ^a^ | | | 0.39±0.27 ^a,b^ | | 0.53±0.25 ^a,b,c^ | | <0.0001 |
|  |  | | |  | | | |  | |
|  | **NORMAL** | | **OVERWEIGHT** | | | **OBESITY** | | **p-value** | |
| BMI | 0.17±0.26 | | 0.33±0.26 ^a^ | | | 0.30±0.28 ^a^ | | <0.0001 | |

Data are reported as mean±SD (Standard Deviation). One-way ANOVA was performed for comparisons among more than two groups, while Student T-test was performed for comparisons between two groups. Statistical significance was assessed for p-values (p) <0.05. ^(a)^ indicates significance vs subjects of the first column; ^(b)^ indicates significance vs subjects of the second column; ^(c)^ indicates significance vs subjects of the third column.

Visceral obesity was diagnosed for Waist Circumference values ≥80 cm in females and ≥94 cm in males. Type 2 Diabetes was diagnosed for FPG >126 mg/dl or HbA1c >6.4% or ongoing anti-diabetic treatment. Metabolic Syndrome was diagnosed when subjects had increased waist circumference plus at least two other criteria among hyperglycaemia, low HDL, hypertriglyceridemia, and hypertension. Hypertension was identified as systolic arterial blood pressure (SAP) ≥130mmHg, diastolic arterial blood pressure (DAP) ≥85mmHg and/or treatment with antihypertensive agents. Liver steatosis was assessed through Abdomen Ultrasound. Mild steatosis is represented by a mild diffuse increase in fine echoes in the hepatic parenchyma with normal visualisation of the diaphragm and intrahepatic vessel borders. Moderate steatosis is represented by a moderate diffuse increase in fine echoes with slightly impaired visualisation of the intrahepatic vessels and diaphragm. Severe steatosis is represented by a marked increase in fine echoes with poor or no visualisation of the intrahepatic vessel borders, diaphragm, and posterior portion of the right lobe of the liver. BMI=25-29.9 Kg/sqm depicts a condition of overweight, while BMI ≥30 Kg/sqm a condition of obesity.

Abbreviations: BMI, Body Mass Index.
